# Simulation Study on Molecular Adsorption of Coal in Chicheng Coal Mine

**DOI:** 10.3390/molecules28083302

**Published:** 2023-04-07

**Authors:** Jingxue Yan, Baoshan Jia, Baogang Liu, Jinyi Zhang

**Affiliations:** 1 College of Safety Science and Engineering, Liaoning Technical University, Fuxin 123000, China; 2 Key Laboratory of Mine Thermal Power Disaster and Prevention, Liaoning Technical University, Ministry of Education, Fuxing 123000, China

**Keywords:** coalbed methane, coal macromolecular structure model, molecular simulation, adsorption properties, microscopic mechanism

## Abstract

To study the importance of the adsorption mechanism of methane (CH_4_) and carbon dioxide (CO_2_) in coal for coalbed methane development, we aimed to reveal the influence mechanism of adsorption pressure, temperature, gas properties, water content, and other factors on gas molecular adsorption behavior from the molecular level. In this study, we selected the nonsticky coal in Chicheng Coal Mine as the research object. Based on the coal macromolecular model, we used the molecular dynamics (MD) and Monte Carlo (GCMC) methods to simulate and analyze the conditions of different pressure, temperature, and water content. The change rule and microscopic mechanism of the adsorption amount, equal adsorption heat, and interaction energy of CO_2_ and CH_4_ gas molecules in the coal macromolecular structure model establish a theoretical foundation for revealing the adsorption characteristics of coalbed methane in coal and provide technical support for further improving coalbed methane extraction.

## 1. Introduction

Coalbed methane (CBM) is a valuable energy source with many advantages such as richness, safety, and environmental protection [1]. China is a large coal-mining country, and coal seam gas resources in storage are very rich. According to relevant statistics in the literature, the depth of more than 2000 m of coal seam gas reserves is about 30.05 × 1012 m^3^. In the coal seam with a depth greater than 2000 m, the reserves of coalbed methane are about 40.71 × 1012 m^3^ [2]. Therefore, efficient and reasonable CBM development is an important way to ensure the security and sustainable development of the national energy strategy. Because CBM usually exists on the surface of the coal body in the form of adsorption state, research on the adsorption characteristics of methane (CH_4_), carbon dioxide (CO_2_), and other gases in the coal seam is the key to solve the CBM development “bottleneck” problem.

In recent years, many researchers have conducted a lot of research on the adsorption characteristics of CH_4_, water (H_2_O), and CO_2_ in coal. Lu et al. [3] conducted adsorption and strain experiments on coal samples at 30 °C, 40 °C, and 50 °C and at a pressure of 15 MPa, which showed that the shapes of swelling strain curves of different grades of coal were similar. Shen et al. [4] used the volumetric method to carry out the high-pressure adsorption experiment, which showed that the adsorption isotherm was consistent with the Langmuir model in the range of pressure and temperature. Wang et al. [5] studied the influence of water, pH value, and coal rank on the adsorption of CO_2_ by coal and showed that water inhibited the adsorption of CO_2_ by coal, and the change of pH value promoted the adsorption of CO_2_ by coal. Qiu et al. [6] conducted an isothermal adsorption experiment on coals with different coal grades, water content, and grain size and showed that the Langmuir volume (V-L) of coal was unrelated to grain size but was inversely proportional to water content. It first decreased and then increased with the increase in coal grade. Ren et al. [7] studied the influence of high temperature and high pressure on methane adsorption and thermal stability of coal of different ranks and found that methane adsorption decreased sharply with an increase in temperature. Weniger et al. [8] conducted CH_4_ and CO_2_ high-pressure adsorption experiments on different coal samples from the Silesia Basin of the Czech Republic and showed that the adsorption capacity had a weak positive correlation with coal rank and a negative correlation with temperature. Zhao Zhigen [9] tested the gas isothermal adsorption capacity of coal samples at 30–70 °C and found that the adsorption constant gradually decreased with an increase in temperature and finally tended to be stable. Zhong Lingwen [10] identified a linear increase relationship between the amount of gas adsorbed by coal and the temperature. Low temperature and low pressure play a leading role, and high temperature and high pressure also play a leading role. These experiments proved that the adsorption mode of coal for CH_4_, H_2_O, and CO_2_ belongs to solid-gas physical adsorption [11], which is easily affected by moisture, temperature, pressure, and coal grade. In the current body of research, however, few studies have examined the influence rules and microscopic mechanism of temperature, water content, gas properties, and adsorption pressure on the adsorption properties of the coal macromolecular model during the adsorption of gas molecules from the molecular level.

Therefore, in this study, we constructed a macromolecular structure model of low-metamorphic nonsticky coal through characterization testing and analysis and studied the pore distribution characteristics of gas-accessible pores in the coal body. We used molecular simulation software. The study of the influential rule and microscopic mechanism of different temperature, pressure, and water content on the adsorption performance of CO_2_ and CH_4_ gas by coal holds good social and economic significance. In addition, the use of the Grand Canonical Monte Carlo (GCMC) and molecular dynamics (MD) methods provides an important basis for gas extraction, CBM resource evaluation, and CO_2_ geological storage.

## 2. Structural Characterization and Construction of Macromolecular Structure of Coal

**Figure 1:**
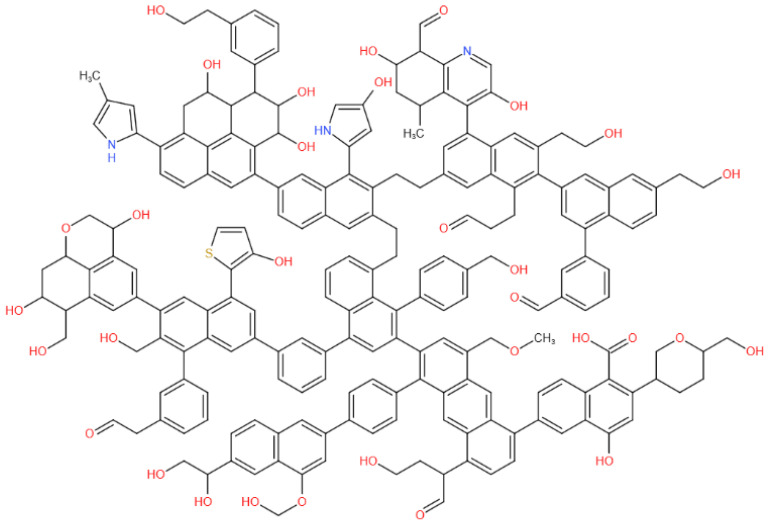
Plane model of coal macromolecular structure.

The study of the physicochemical structure of coal can enable a complete understanding of the adsorption performance of coal for gas [12]. In this study, we selected fresh coal samples (density 1.16 g/cm^3^, *R*°_max_ 0.665%) from the 1502-2 working face of Chicheng Coal Mine. The coal samples were crushed, screened, and divided by a crusher and vibrating screen machine to produce analytical samples with a particle size below 200 mesh. Based on the results of elemental analysis, Fourier-transform infrared spectroscopy (FT-IR), X-ray photoelectron spectroscopy (XPS), and carbon-13 nuclear magnetic resonance (^13^C NMR) experimental characterization, we determined the molecular formula of nonsticky coal in Chicheng Coal Mine to be C_207_H_181_O_32_N_3_S (C: 76.39%, N: 1.29%, O: 15.73%, H: 5.61%, S: 0.99). The coal macromolecular model is shown in Figure 1.

## 3. Theoretical Analysis of Molecular Simulation of Gas Adsorption in Coal

### 3.1. Selection of Force Field

In this study, we used MD and GCMC methods to simulate the adsorption mechanism of CO_2_ and CH_4_ gas molecules in coal macromolecular models under different conditions. We selected the COMPASS force field for model optimization and adsorption simulation, which fully considered the interactions between molecules. The parameters were derived from ab initio parameterization and empirical optimization [13].

### 3.2. Transformation of Pressure and Fugacity

For nonideal gas, fugacity and pressure are different at different temperatures. The higher the pressure, the greater the difference between fugacity and pressure. In this study, we adopted the Peng–Robinson equation of state to calculate fugacity and pressure conversion of nonideal gas, with specific steps as follows [14]:

(1) $$P=\frac{RT}{V-b}-\frac{a\left(T\right)}{V\left(V+b\right)+b\left(V-b\right)}$$

Then, Equation (1) can be rewritten as follows:

(2) $$Z^{3}-\left(1-B\right)Z^{2}+\left(A-3B^{2}-2B\right)Z-\left(AB-B^{2}-B^{3}\right)=0$$

where *Z* is the gas compression factor, and *A* and *B* are the equation coefficients, which can be expressed as follows [13]:

(3) $$A=\frac{aP}{R^{2}T^{2}}$$

(4) $$B=\frac{bP}{RT}$$

(5) $$Z=\frac{PV}{RT}$$

where *P* is the gas pressure, MPa; *R* is the mole constant of gas; and *V* is the molar volume of the gas, 22.4 L/mol.

Next, Equation (1) is Equations (6) and (7) [13] at the critical point and Equations (8) and (9) at the noncritical point [13]:

(6) $$a\left(T_{c}\right)=0.45724\frac{R^{2}T_{c}^{2}}{P_{c}}$$

(7) $$b\left(T_{c}\right)=0.07780\frac{RT_{c}}{P_{c}}$$

(8) $$\begin{array}{l} a\left(T\right)=a\left(T_{c}\right)\cdot a\left(T_{r},\omega \right)\\ =0.45724\frac{R^{2}T_{c}^{2}}{P_{c}}{\left[1+\left(0.37464+1.54226\omega -0.26992\omega^{2}\right)\left(1-\sqrt{\frac{T}{T_{c}}}\right)\right]}^{2} \end{array}$$

(9) $$b\left(T\right)=b\left(T_{c}\right)=0.07780\frac{RT_{c}}{P_{c}}$$

where *T_r_* is the comparison temperature, K, and *T_r_ = T/T_c_*; *T_c_* is the critical temperature, K; *p_c_* is the critical pressure, MPa; and *ω* is the eccentricity factor.

By substituting Equation (10) into Equation (1), the fugacity (f) of a single component gas can be derived as follows:

(10) $$\ln\frac{f}{P}=\int_{0}^{P}\left(\frac{V}{RT}-\frac{1}{P}\right)dP$$

(11) $$\ln\frac{f}{P}=Z-1-\ln\left(Z-B\right)-\frac{A}{2\sqrt{2}B}\ln\left(\frac{Z+2.414B}{Z-0.414B}\right)$$

According to these principles, the conversion between fugacity and pressure of different gases at different temperatures can be performed using Perl script in Materials Studio 2017 (MS) [15].

## 4. Molecular Simulation of Gas Adsorption Characteristics of Coal

Based on the macromolecular structure model of noncohesive coal in the Chicheng Coal Mine, we studied the adsorption microscopic properties of CH_4_ and CO_2_ molecules in the macromolecular model by using GCMC and MD in molecular simulation [16].

### 4.1. Adsorption Model Construction and Optimization

#### 4.1.1. Coal Model Construction and Optimization

**Figure 2:**
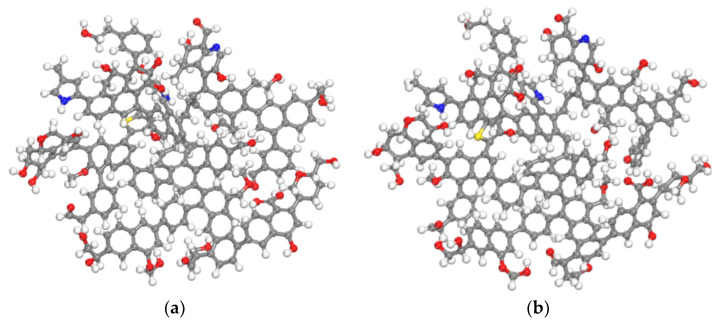
Comparison before and after model optimization. (**a**) Initial model and (**b**) optimization model.

We imported the two-dimensional plane model of coal macromolecules constructed in Figure 1 into MS (Materials Studio 2017, Accelrys, San Diego, CA, USA) molecular simulation software and constructed the initial three-dimensional structure, as shown in Figure 2a. We used the Forcite module for geometric optimization of the model and selected the COMPASS force field for geometric optimization. We set charges as assigned by Forcefield, calculation accuracy as Fine, and iteration steps as 5000. Then, we annealed the model and selected the NVT family. For Nose, the temperature was 300–600 K, and the number of cycles was five. The aerodynamic parameters were set as specified. The model structure after dynamic optimization is shown in Figure 2b.

The energy changes of the macromolecular structure model of coal before and after optimization are shown in Table 1. As shown in Table 1, after optimization of the initial model, the valence electron energy and nonbond energy of the coal molecules both decreased somewhat. In the final model, the valence electron energy of coal molecules was higher than the nonbond energy, which was the main part of the total energy and contributed more to the stability of the model, and the bond torsion energy played a major role in valence electron energy. The chemical bond torsion of coal molecules was the basis for the model to bend and twist into a stereoscopic configuration. In the nonbonding energy, van der Waals energy was dominant.

**Figure 3:**
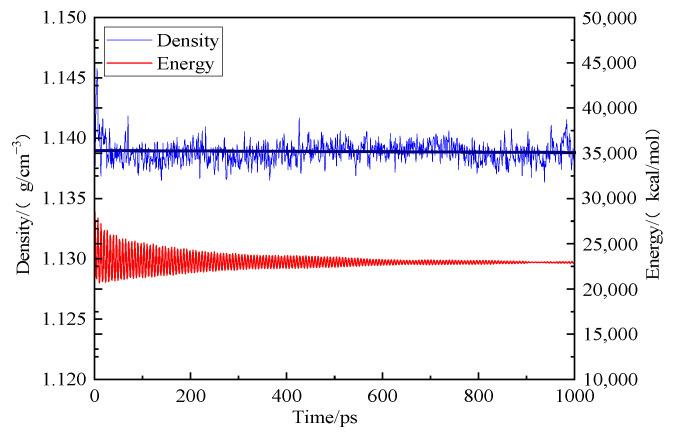
Trends of energy and concentration of different coal samples during kinetic optimization.

To establish the periodic boundary condition, we used the Amorphous Cell module to put 10 optimized coal molecular models into the periodic cell. First, we conducted the geometric optimization, and then, we subjected the model to the 300–600 K NPT system annealing treatment. A total of five cycles were set up. We used the COMPASS force field, the atom-based method for the van der Waals term, and the Ewald method for the electrostatic action term. After annealing and kinetic treatment for 1000 ps, the total energy of the coal cell model was reduced to the lowest level and then stabilized at 22,985.040 kcal/mol. The density of the coal cell structure was stabilized at 1.138 g/cm^3^, which was close to the real density of coal, as shown in Figure 3.

**Figure 4:**
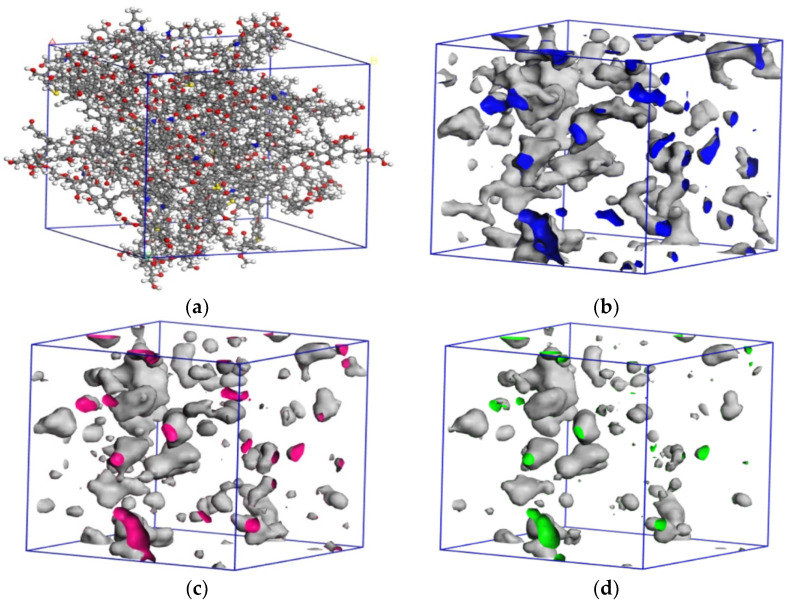
Coal macromolecular structure cell model and pore size distribution of different gases. (**a**) Coal macromolecular structure cell model, (**b**) He micropore distribution, (**c**) pore distribution of CO_2_ micropores, and (**d**) pore distribution of CH_4_ micropores.

The optimized coal macromolecular structure model is shown in Figure 4a, whose molecular formula is C_2070_H_1810_N_30_O_320_S_10_. By using Atom Volumes and Surfaces with the MD radii of helium (He) (0.13 nm), CO_2_ (0.165 nm), and CH_4_ (0.19 nm) as the Connolly probe radii, we calculated the pore profiles of different gases in the coal molecular surfaces. As shown in Figure 4a–c, the accessible hole of the He gas in the model was 3071.15 Å^3^, the accessible hole of CO_2_ was 2295.89 Å^3^, and the accessible hole of CH_4_ was 1550.92 Å^3^. The pore space on the coal surface was the main place for gas adsorption. The CO_2_ was absorbed more easily by the coal surface than CH_4_ in terms of the adsorption space provided by the coal surface for gas.

#### 4.1.2. Construction and Optimization of Adsorbent Model

**Figure 5:**
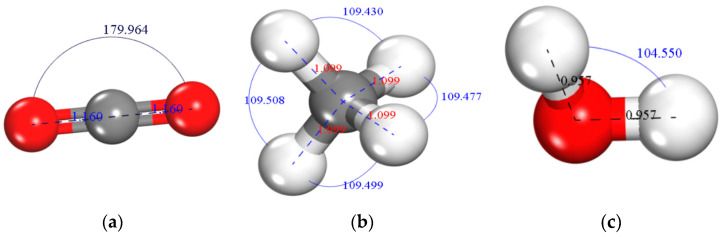
Small molecule model for different gas sorbents. (**a**) CO_2_, (**b**) CH_4_, and (c) H_2_O.

We used MS software to visualize and optimize the gas molecules, H_2_O, CO_2_, and CH_4_. The energy changes of the three optimized gas molecules and the final models are shown in Table 2 and Figure 5.

### 4.2. Simulation Methods and Parameter Settings

We used the Sorption module in the MS software to study the coal adsorption characteristics. We analyzed the adsorption behavior of CO_2_ and CH_4_ gas molecules in dry and water-containing coal macromolecular structure models according to the GCMC method. We performed adsorption simulation using fixed pressure in the Sorption module. The calculation accuracy was customized with equilibration steps and production steps of 1,000,000. We selected the COMPASS force field. We set the charges as assigned by Forcefield, the electrostatic action term as determined by the Ewald method, and the van der Waals energy according to an atom-based method. The adsorption equilibrium condition prevailed when the C/D ratio in the result file was close to one.

The unit of adsorption capacity in the simulation is molecules/u.c., and the unit of adsorption capacity in engineering is mL/g, which needs to be converted as follows:

(12) $$Q=\frac{N}{M}$$

where *Q* is the gas adsorption capacity, mol/g; *N* is the number of adsorbed gases, molecules/u.c.; and *M* is the molar mass of coal molecular cell, g/mol.

The gas adsorption capacity calculated in the software was the absolute adsorption capacity of gas. In engineering applications, however, capacity usually is the excess adsorption capacity of gas. A previous study [12] noted that the gas molecular adsorption quantity of the excess adsorption capacity was equivalent to the absolute adsorption capacity of coal minus the gas adsorption quantity contained in the pore volume of the coal macromolecular structure under simulated pressure and temperature, which can be expressed as follows [12]:

(13) $$N_{ex}=N_{ab}-N_{A}PV_{v}/RT$$

where *N_ex_* is the amount of excess adsorption capacity of gas molecules in the coal macromolecular structure, molecules/u.c.; *N_ad_* is the number of absolute adsorption capacity of gas molecules in the coal macromolecular structure, molecules/u.c.; *N_A_* is Avogadro’s constant, which is 6.02 × 10^23^; and *V_v_* is the accessible pore capacity in the macromolecular structure of coal, mL.

The excess adsorption capacity of gas molecules adsorbed by the coal macromolecular structure model was converted to the adsorption capacity, mL/g, under the following standard conditions:

(14) $$Q_{ex}=22,400\times \frac{N_{ex}}{M}$$

where *Q_ex_* is the excess adsorption capacity of coal macromolecular structure model (mL/g).

### 4.3. Correctness Verification of the Model

**Figure 6:**
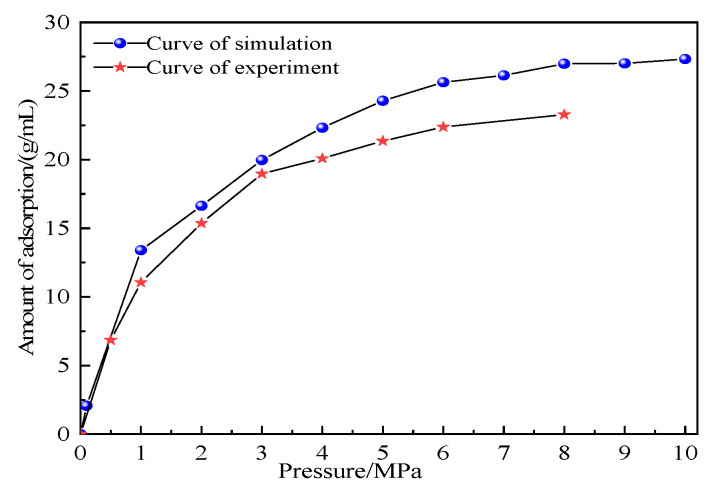
Simulation and experimental comparison of adsorption capacity of CH_4_.

In this study, we verified the experimental and simulated adsorption isotherms of CH_4_, as shown in Figure 6, under the following conditions: temperature = 25 °C and pressure = 0–8 MPa. (Note that the experimental data came from the Akagi Coal Mine.) As shown in Figure 6, although our simulation results were slightly higher than the experimental results, they were in good agreement, which may have been because the coal macromolecule model considered only the organic part and ignored the inorganic part. A comparative analysis can prove that the relevant parameters established in this study are reasonable.

### 4.4. Influence of Temperature on Adsorption Characteristics

#### 4.4.1. Influence of Temperature on Adsorption Capacity

**Figure 7:**
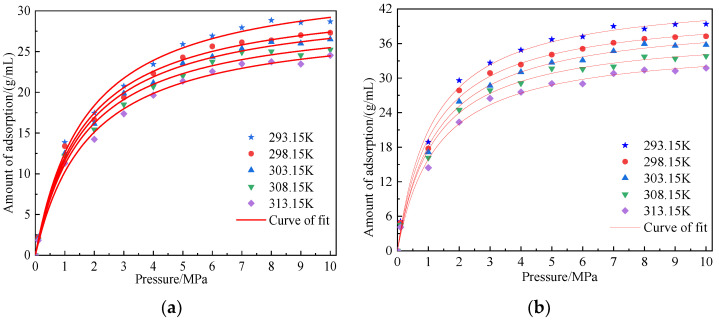
Adsorption curves of CH_4_ and CO_2_ on coal macromolecular models at different temperatures. (**a**) Coal-CH_4_ adsorption curve and (**b**) coal-CO_2_ adsorption curve.

The isothermal adsorption curve of coal can be used to predict the recoverable amount of CBM. We evaluated the sealing capacity and saturation state of CBM [17]. Figure 7 shows the adsorption isotherm of CO_2_ and CH_4_ in the coal macromolecule model at temperatures of 293.15 K, 298.15 K, 303.15 K, 308.15 K, and 313.15 K and pressures of 100–10 MPa. We used the Langmuir model to fit the adsorption structure of CO_2_ and CH_4_ gas in coal. The expression is shown in Equation (15), and the fitting results are shown in Table 3.

(15) $$Q=\frac{abP}{1+bP}$$

where *a* is the saturated adsorption capacity of gas, mL/g, and *b* is the reciprocal of Langmuir pressure, MPa^−1^.

The simulation results showed the following: When the adsorption temperature was the same, the adsorption capacity of CO_2_ and CH_4_ increased with an increase in pressure. When the pressure was low, the adsorption capacity of coal to gas increased rapidly. When the pressure was greater than 5 MPa, the adsorption isotherm of coal gas tended to be gentle. This trend of CO_2_ adsorption isotherm was more obvious than that of CH_4_, indicating that the CO_2_ adsorption capacity of coal tended to saturate faster under the same pressure. Within the simulated temperature and pressure range, the saturated adsorption capacity of CO_2_ was 1.24–1.31 times that of CH_4_, which was mainly due to the difference in the MD diameter, critical pressure, boiling point, and polarizability of the two gases. When the adsorption pressure was the same, the adsorption capacity of the two gas molecules decreased with the increase in temperature, indicating that the increase in temperature was not conducive to the adsorption of gas molecules by the coal macromolecule model. The increase in temperature promoted the increase in energy, activity, and kinetic energy of gas molecules, which was not conducive to the “capture” of gas molecules by the coal molecular surface during the adsorption process. Moreover, high temperature inhibited the transformation of gas molecules from the free state to the adsorption state, and some stable adsorbed gases also experienced desorption into active free-state gases because of this high temperature. Therefore, the adsorption capacity of coal to gas would decrease with the increase in temperature. This result showed that the adsorption of CH_4_/CO_2_ by coal samples was an exothermic reaction.

**Figure 8:**
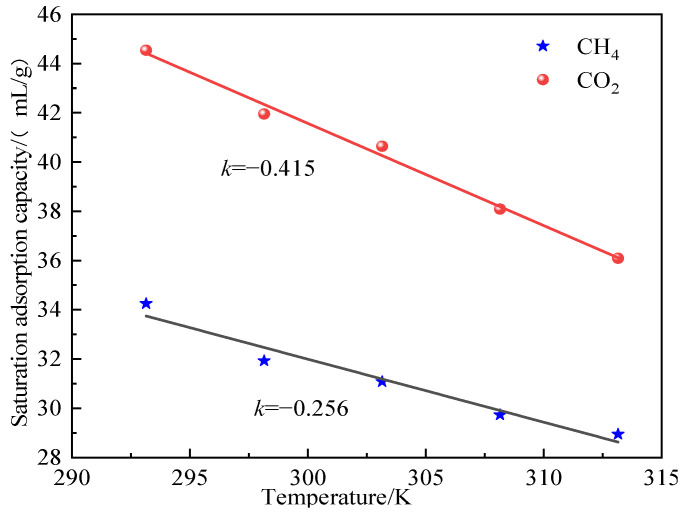
Saturated adsorption amount of CO_2_ and CH_4_ gas fitting curve along with the change of temperature.

As shown in Figure 8, the absolute value of slope *k* of CO_2_ gas was greater than that of CH_4_, indicating that the influence of temperature change on the adsorption amount of CO_2_ was greater than that of CH_4_, and the inhibition degree of the adsorption amount of CO_2_ was stronger at high temperature. This result was related mainly to the interaction between the two gases and the functional groups on the coal surface.

**Figure 9:**
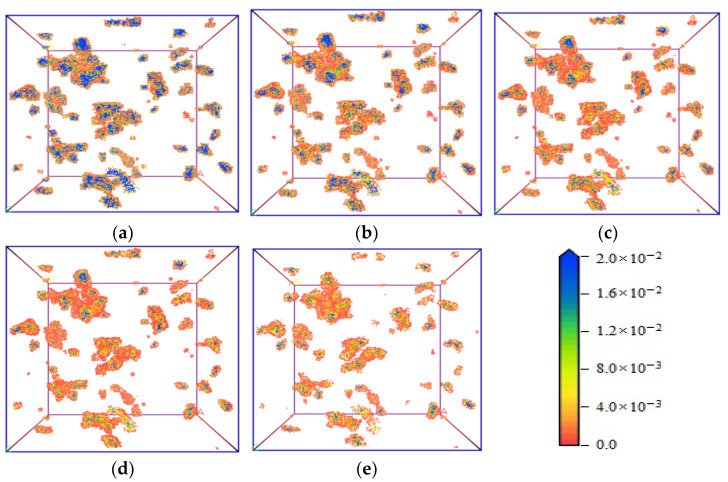
Density distribution characteristics of CO_2_ at different temperatures. (**a**) 293.15 K, (**b**) 298.15 K, (**c**) 303.15 K, (**d**) 308.15 K, and (**e**) 313.15 K.

**Figure 10:**
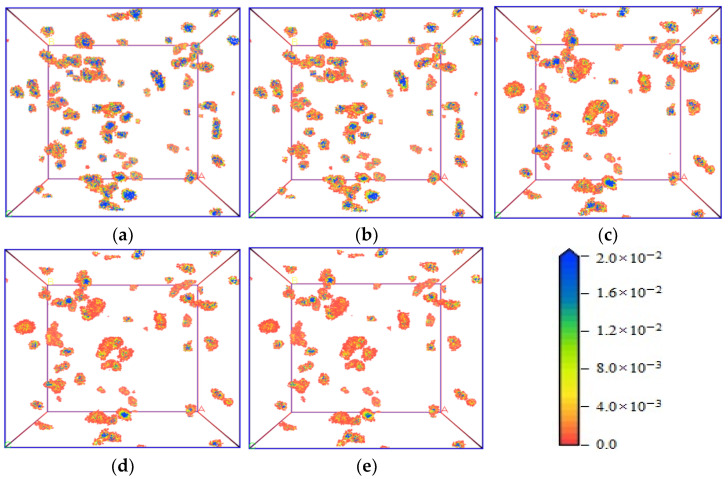
Density distribution characteristics of CH_4_ at different temperatures. (**a**) 293.15 K, (**b**) 298.15 K, (**c**) 303.15 K, (**d**) 308.15 K, and (**e**) 313.15 K.

Figure 9 and Figure 10 show the gas density distribution characteristics of CO_2_ and CH_4_ adsorbed by the coal macromolecular structure model under an adsorption pressure of 5 MPa and at different temperatures. With the increase in temperature, the adsorption sites of the two gases were almost the same, and we did not observe any particularly significant differences. The density of the adsorbed gas, however, decreased with the increase in temperature, which was more obvious from the comparison of the adsorption simulation results of the two gases at 293.15 K and 313.15 K. The simulation results of these two temperatures also showed that the space size and quantity of gas at the adsorption sites in the coal molecular structure at low temperature slightly increased compared with that at high temperature, and the density of gas adsorbed at the adsorption sites was also higher, which resulted in a larger amount of gas adsorption. At the same temperature, most of the adsorption sites of the two gases remained the same, but the space and density of the adsorption sites of CO_2_ were greater than that of CH_4_.

#### 4.4.2. Influence of Temperature on Adsorption Heat

In the study of the gas adsorption behavior law of coal, adsorption heat is often used to judge the physical and chemical nature of CBM adsorption by coal rocks, which holds great significance to explain the adsorption law and adsorption mechanism [18]. Based on the energy particle fluctuation calculation in the great canonical ensemble [19], the equivalent adsorption heat *Q_st_* in the adsorption process of coal and gas can be obtained as follows:

(16) $$Q_{st}==RT-\left[\frac{\partial \left(U_{total}-U_{intra}\right)}{\partial N_{total}}\right]$$

where *U_total_* is the total interaction energy in the system, kJ/mol, and *U_intrl_* is the internal energy of the gas molecule, kJ/mol.

**Figure 11:**
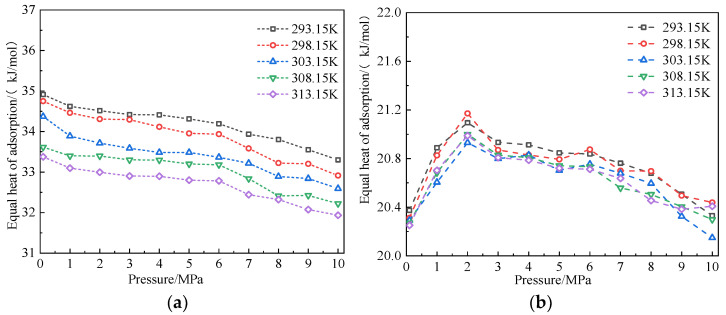
Variation law of adsorption heat of CO_2_ and CH_4_ in coal at different temperatures. (**a**) CO_2_ and (**b**) CH_4_.

Figure 11 shows the variation law of equal adsorption heat of CO_2_ and CH_4_ in coal within the range of simulated temperature and pressure. The simulation results showed that under the same pressure condition, the equivalent adsorption heat of CO_2_ gradually decreased with the increase in temperature, whereas the equivalent adsorption heat of CH_4_ changed in a small range [20]. This result indicated that the change in temperature had a small impact on the equivalent adsorption heat of the coal-CH_4_ system. Under the same temperature condition, the equivalent adsorption heat of CO_2_ gradually decreased with the increase in pressure, and the equivalent adsorption heat of CH_4_ increased with the increase in pressure at 0–2 MPa and then decreased with the increase in pressure. The equal adsorption heat was jointly affected by two factors: the interaction between gas and coal, and the interaction between gas fractions [14]. Obviously, the CO_2_-coal interaction was dominant in the process of adsorption pressure change. For CH_4_, the interaction between gas fractions was dominant at a low pressure, whereas the interaction between gas and coal was dominant at a high pressure. In the adsorption process, the equivalent adsorption heat ranges for CO_2_ and CH_4_ were 31.93–34.91 kJ/mol and 20.60–20.88 kJ/mol, respectively. The equivalent adsorption heat of CO_2_ was greater than that of CH_4_ because CO_2_ had the highest polarizability. Moreover, it had the highest quadrupole moment and the smallest MD diameter. Compared with CH_4_, there was a stronger interaction between CO_2_ and the coal surface, which resulted in the maximum heat release from the coal-adsorbed CO_2_. The equivalent heat of adsorption of the system reflected the adsorption capacity of coal to gas to some extent, but the variation trend of the equivalent heat of adsorption and the adsorption amount was not completely the same. For example, the equivalent heat of adsorption of CH_4_ at 298.15 K was greater than that of CH_4_ at 293.15 K, but the adsorption amount followed the opposite trend. Since the equivalent adsorption heat of the two gases in coal was less than 42 kJ/mol, the adsorption of CO_2_ and CH_4_ in coal was physical adsorption [13].

#### 4.4.3. Influence of Temperature on Interaction Energy

By calculating the interaction energy between gas and coal in the system, we evaluated the interaction strength between coal and gas [21]. The lower the calculated energy, the greater the absolute value of the interaction energy-that is, the more stable the adsorption between the two. The interaction energy can be calculated as follows:

(17) $$E_{abs}=E_{A/B}-\left(E_{A}+E_{B}\right)$$

where *E_A/B_* is the total energy of the system, kJ/mol; *E_A_* is the energy of coal model, kJ/mol; and *E_B_* is the energy of the gas molecule, kJ/mol.

**Figure 12:**
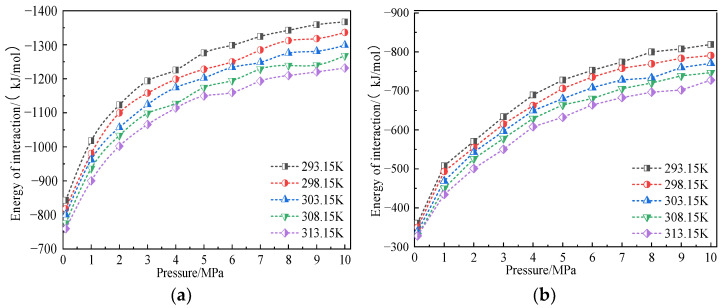
Variation law of interaction energy of CO_2_ and CH_4_ in coal at different temperatures. (**a**) CO_2_ and (**b**) CH_4_.

We further studied the influence of temperature on the interaction energy between coal and gas. Figure 12 shows the variation curve of interaction energy between CH_4_/CO_2_ gas molecules and coal macromolecular structure model at temperatures of 293.15 K, 298.15 K, 303.15 K, 308.15 K, and 313.15 K and pressures of 100 kPa–10 MPa.

The simulation results showed that, under the same temperature condition, the interaction energy between coal, CH_4_, and CO_2_ increased with an increase in adsorption pressure, which was consistent with the trend of adsorption capacity changing with pressure. Under the same adsorption pressure, the interaction energy between coal and CH_4_ and CO_2_ decreased with the increase in temperature, which was consistent with the trend of adsorption capacity changing with temperature. These results indicated that the interaction energy between coal and gas in the system is the essential factor determining the adsorption capacity.

In the simulated temperature and pressure range, the interaction energy between coal and CO_2_ ranged from −759.51 kJ/mol to −1367.25 kJ/mol, the interaction energy between coal and CH_4_ ranged from −328.01 kJ/mol to −819.15 kJ/mol, and the interaction energy between coal and CO_2_ was greater than that of CH_4_. It was about 1.67–2.31 times that of CH_4_. These research results revealed the influence mechanism of temperature, pressure, and gas properties on gas adsorption on coal surface from the perspective of energy-that is, a stronger interaction energy led to a larger adsorption capacity.

**Figure 13:**
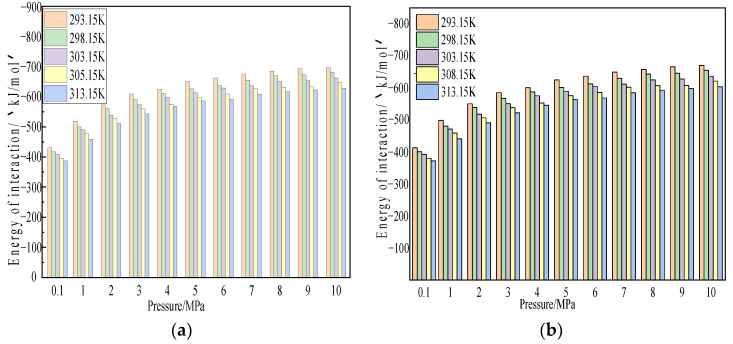
Variation characteristics of van der Waals energy and electrostatic energy of CO_2_ under different temperature and pressure conditions. (**a**) Variation characteristics of CO_2_ van der Waals and (**b**) variation characteristics of CO_2_ electrostatic energy.

**Figure 14:**
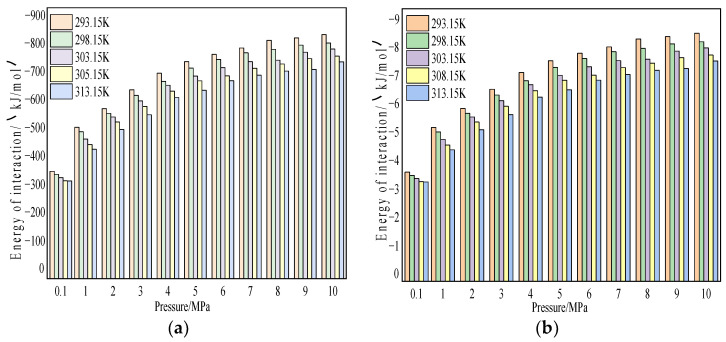
Variation characteristics of van der Waals energy and electrostatic energy of CH_4_ under different temperature and pressure conditions. (**a**) CH_4_ van der Waals variation characteristics and (**b**) variation characteristics of CH_4_ electrostatic energy.

The interaction energy was composed of van der Waals energy and electrostatic energy [22]. Figure 13 and Figure 14 show the characteristics of the van der Waals interaction energy and electrostatic energy between coal and CH_4_/CO_2_ as a function of temperature and pressure. The simulation results showed that with the increase in pressure, the van der Waals energy and electrostatic energy of coal and CH_4_/CO_2_ gradually increased. With the increase of temperature, the two forms of energy in the system gradually weakened. In the adsorption process, the van der Waals energy of coal and CO_2_ accounted for about 51%, and the electrostatic energy accounted for about 49%. In contrast, the van der Waals energy of coal and CH_4_ accounted for about 99%, and the electrostatic energy accounted for about 1%. This result showed that the van der Waals interaction energy played a dominant role in the adsorption, in particular for CH_4_, because the electrostatic interaction is a long-range interaction with an action range of only a few nanometers. Although the simulation model satisfied the distance condition, the number of atoms with different charges in the coal and gas molecules was small, and therefore, the charge difference between them was also small [22].

### 4.5. Influence of Moisture on Adsorption Characteristics

In the development process of CBM mining, the influence of water content in coal on gas adsorption cannot be ignored. The relevant literature has indicated that the content of water in coal can generally reach about 8 wt.% [21,22,23,24]. We used the molecular simulation method to study the influence of water in coal on the adsorption of gas in coal and its mechanism.

#### 4.5.1. Water-Bearing Coal Model Construction and Pore Analysis

**Figure 15:**
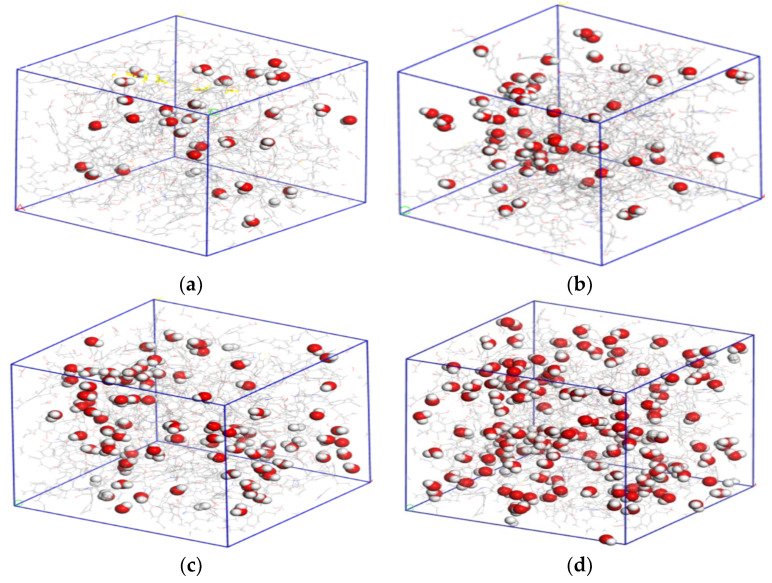
Molecular models of coal with different moisture content. (**a**) 1% moisture content, (**b**) 2% moisture content, (**c**) 3% moisture content, and (**d**) 5% moisture content.

According to the actual situation of water cut in Chicheng Coal Mine’s coalbed, we established the macromolecular structure models of nonsticky coal with water cuts of 1%, 2%, 3%, and 5%. According to the calculation, the number of water molecules in the different models with moisture content of 1%, 2%, 3%, and 5% was 31, 63, 94, and 156, respectively. The macromolecular models of coal with different water cuts are shown in Figure 15.

**Figure 16:**
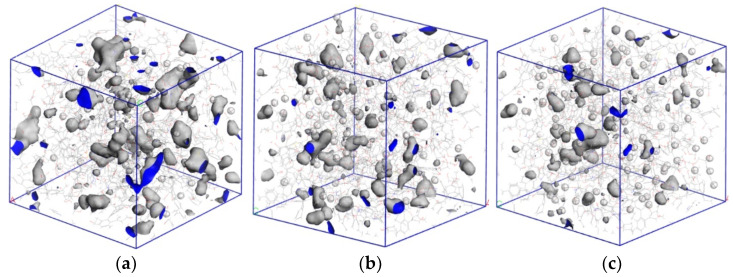
Pore characteristics of CO_2_ with different moisture content that can enter the pore. (**a**) 1% moisture content, (**b**) 2% moisture content, and (**c**) 3% moisture content.

**Figure 17:**
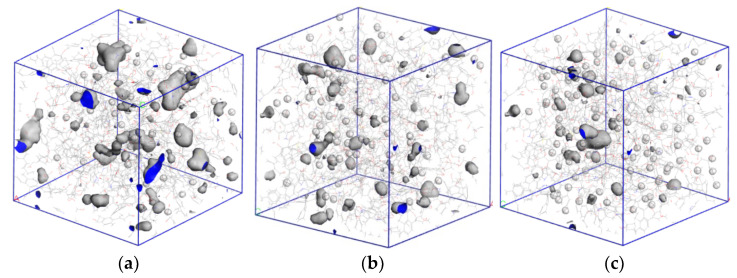
Pore characteristics of CH_4_ with different moisture content that can enter the pore. (**a**) 1% moisture content, (**b**) 2% moisture content, and (**c**) 3% moisture content.

The presence of water in coal samples had a significant influence on the pore distribution of coal. To further study the influence of water content in coal on the adsorption and diffusion effect of CO_2_ and CH_4_ gas, we used MS software to simulate the pore characteristics of CO_2_ and CH_4_ in wet coal with water content of 1%, 2%, 3%, and 5%. The simulation results showed that if the MD radius of CO_2_ was taken as the probe radius, the accessible pore volumes of wet coal with 1%, 2%, 3%, and 5% moisture content were 1719.29 Å^3^, 973.92 Å^3^, 693.69 Å^3^, and 337.96 Å^3^, respectively. Compared with the accessible pore volumes of the dry coal sample (2295.89 Å^3^), the volume decreased by 25.11%, 57.58%, 69.78%, and 85.27%, respectively. Taking the MD radius of CH_4_ as the probe radius, the pore volume of wet coal with 1%, 2%, 3%, and 5% moisture content was 1044.98 Å^3^, 460.71 Å^3^, 321.41 Å^3^, and 127.96 Å^3^, respectively. Compared with the pore volume of the dry coal sample (1550.92 Å^3^), the effect of the increase in water content on CH_4_ gas with a large MD radius was greater than that of CO_2_ gas. Figure 16 and Figure 17 show the pore characteristics of CO_2_ and CH_4_ accessible pores in coal samples with water content of 1%, 2%, and 3%. The simulation results showed that the presence of water in coal greatly reduced the accessible pore volume of the two gases in coal. The higher the water content of coal, the smaller the accessible pore volume of the two gases-that is, the stronger the inhibition on gas adsorption capacity.

#### 4.5.2. Influence of Moisture Content on Adsorption Capacity

**Figure 18:**
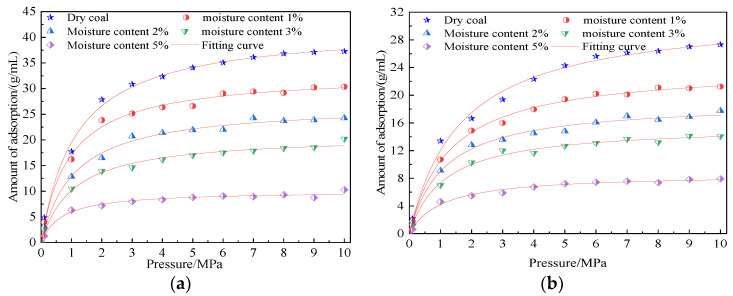
Adsorption curves of CO_2_/CH_4_ on coal macromolecular model at different water content. (**a**) Coal-CO_2_ adsorption curve and (**b**) coal-CH_4_ adsorption increment curve.

At a temperature of 298.15 K and a pressure of 100 kPa–10 MPa, the adsorption isotherms of CO_2_ and CH_4_ gas molecules in the coal macromolecular structure model with water content of 1%, 2%, 3%, and 5% are as shown in Figure 18.

The simulation results showed that the adsorption capacity of the two gases in the water-bearing coal sample increased with the increase of pressure. The adsorption capacity increased rapidly at low pressure and slowly at high pressure, indicating that the moisture in the coal did not change the trend of the adsorption capacity changing with the pressure. The adsorption isotherm of CO_2_ and CH_4_ in water-bearing coal samples satisfied the Langmuir equation. The fitting parameters are shown in Table 4, and the fitting accuracy is above 0.97. Compared with dry coal samples, the adsorption capacity of CO_2_ and CH_4_ gas decreased significantly with an increase in water content. In the adsorption system of coal and CO_2_, the saturated adsorption capacity of coal samples containing 1%, 2%, 3%, and 5% water was 21.43%, 35.73%, 50.18%, and 76.14% lower, respectively. In the adsorption system of coal and CH_4_, the saturated adsorption capacity of coal samples containing 1%, 2%, 3%, and 5% water was 24.43%, 40.36%, 50.08%, and 73.07% lower, respectively, indicating that water in coal was not conducive to the adsorption of CO_2_ and CH_4_ in coal because the existence of water shrank the pore space inside coal. In addition, water had stronger adsorption on the coal surface than CO_2_ and CH_4_, occupying the original adsorption sites of the two gases through competitive adsorption. In addition, water not only had a strong dipole moment but also generated a Coulomb force between water molecules. Therefore, the presence of water reduced the interaction force between coal and gas to a certain extent [21], resulting in a decrease in the adsorption capacity of coal for the two gases.

**Figure 19:**
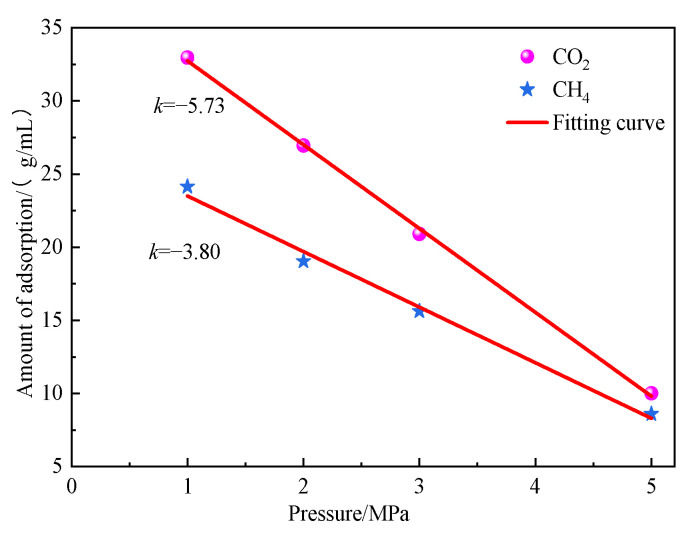
Fitting curves of saturated adsorption capacity of CO_2_ and CH_4_ gas as a function of water content.

As shown in the fitting curve of saturation adsorption capacity of the two gases changing with temperature in Figure 19, the absolute value of slope *k* of CO_2_ was greater than that of slope *k* of CH_4_, which indicated that the change of water content had a greater impact on the adsorption capacity of CO_2_ than on that of CH_4_, and the inhibitory effect of water content on the adsorption capacity of CO_2_ was stronger. According to this analysis, in actual engineering, the coal seam gas extraction effect can be improved through coal seam water injection, such as hydraulic fracturing and other technologies in drilling holes.

#### 4.5.3. Influence of Moisture Content on Adsorption Heat

**Figure 20:**
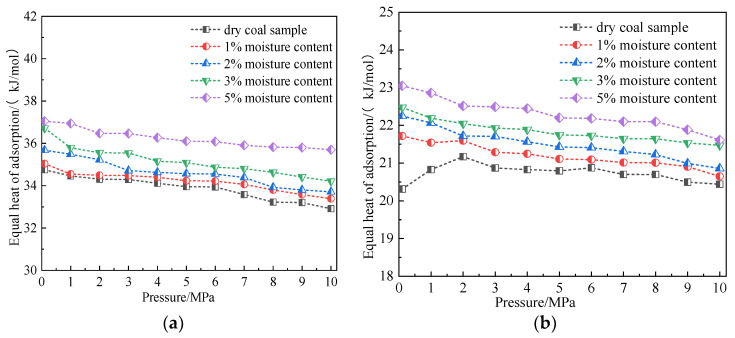
Variation law of adsorption heat of CO_2_ and CH_4_ in coal with different moisture content. (**a**) CO_2_ and (**b**) CH_4_.

To further reveal the influence of water on the gas adsorbed by coal, we calculated the equivalent adsorption heat of CO_2_ and CH_4_ in coal samples with different water content when the temperature was 298.15 K and the simulated pressure was 100 kPa–10 MPa, as shown in Figure 20. The simulation results showed that the equivalent adsorption heat of CO_2_ and CH_4_ was 33.38–37.05 kJ/mol and 20.60–20.88 kJ/mol, respectively. The equivalent adsorption heat of CO_2_ was still greater than that of CH_4_ in the water-bearing coal sample. With the increase of pressure, the equivalent adsorption heat of CO_2_ and CH_4_ in the water-bearing coal sample decreased, which indicated that the interaction between CO_2_/CH_4_ and coal was dominant in this process. The equal adsorption heat of the two gases decreased in the water-bearing coal sample because H_2_O molecules occupied part of the adsorption sites of CO_2_ and CH_4_ on the coal surface. Thus, there were fewer adsorption molecules of CO_2_ and CH_4_ at the strong energy position. Under the same pressure condition, the equivalent adsorption heat of CO_2_ and CH_4_ gradually increased with the increase in water content. Compared with the average equivalent adsorption heat of the dry coal sample in the pressure range (CO_2_:33.88 kJ/mol, CH_4_: 20.72 kJ/mol), the mean isothermal adsorption heat of CO_2_ and coal samples containing 1%, 2%, 3%, and 5% water increased by 0.94%, 2.12%, 3.77%, and 7.02%, respectively. The average equivalent adsorption heat of CH_4_ and coal samples containing 1%, 2%, 3%, and 5% water increased by 2.31%, 3.76%, 5.45%, and 7.67%, respectively. The equivalent adsorption heat of the two gases adsorbed by water-bearing coal samples increased because CO_2_ and water combined to form carbonic acid on the surface of coal, thus releasing heat. As the increase of water in coal continued to promote the reaction of CO_2_ and water, increasingly more heat was released. Although CH_4_ did not react with water chemically, CH_4_ molecules and water molecules formed hydration molecules [21], resulting in the reduction of energy in the system. Therefore, the higher the water content, the more the heat would be released. In addition, the adsorption heat of the two gases on the water-bearing coal sample was less than 42 kJ/mol. Thus, the adsorption of the two gases on the water-bearing coal sample also was physical adsorption.

#### 4.5.4. Influence of Water Content on Interaction Energy

**Figure 21:**
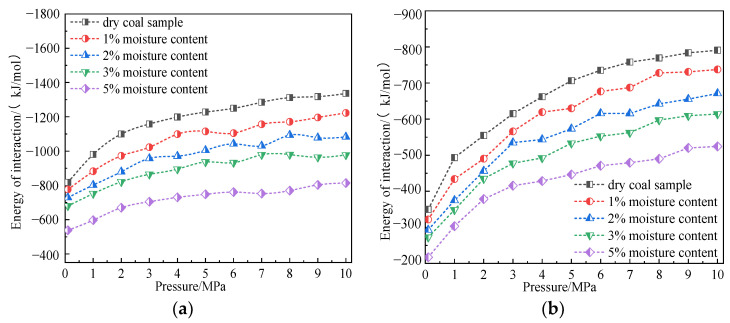
Variation law of interaction energy between CO_2_ and CH_4_ in coal with different moisture content. (**a**) CO_2_ and (**b**) CH_4_.

We further studied the influence of temperature on the interaction energy between coal and gas. Figure 21 shows the interaction energy change curves of CO_2_ and CH_4_ gas molecules in the coal macromolecular structure model with water content of 1%, 2%, 3%, and 5% at a temperature of 298.15 K and a pressure of 100 kPa–10 MPa.

According to the simulation results, under the same water content condition, the interaction between the two gases, wet coal, and CH_4_/CO_2_ increased with the increase of pressure, which was the same as the change trend of dry coal samples with pressure. In the range of simulated pressure, the interaction decreased with the increase in water content, indicating that water could reduce the interaction between gas molecules and coal. The interaction energy of the water-bearing coal sample for CO_2_ adsorption was −538.481 kJ/mol to −1222.61 kJ/mol, and the interaction energy for CH_4_ adsorption was −216.931 kJ/mol to −737.48 kJ/mol. Under the same conditions, the interaction energy of CO_2_ in the system was greater than that of CH_4_, and the interaction energy of water-containing coal samples decreases compared with dry coal samples.

**Figure 22:**
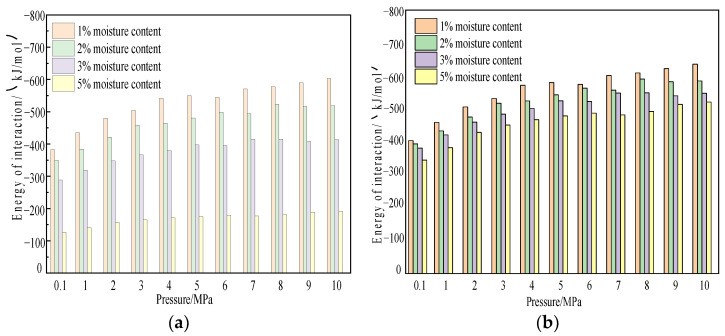
Variation characteristics of van der Waals energy and electrostatic energy of CO_2_ under different water contents. (**a**) CO_2_ van der Waals interaction and (**b**) variation characteristics of CO_2_ electrostatic energy.

**Figure 23:**
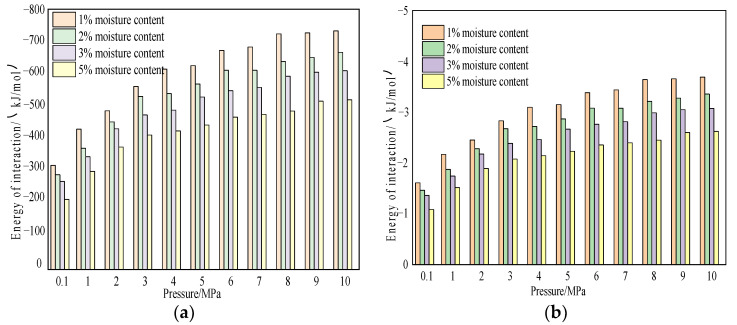
Variation characteristics of van der Waals energy and electrostatic energy of CH_4_ under different water contents. (**a**) CO_2_ van der Waals interaction and (**b**) variation characteristics of CO_2_ electrostatic energy.

Figure 22 and Figure 23 show the variation characteristics of van der Waals energy and electrostatic energy of CH_4_/CO_2_ on the coal surface at different pressures and with different water content during the adsorption process. According to the simulation results, the van der Waals energy and electrostatic energy of the two gases increased with the increase in pressure and decreased with the increase in water content. When CO_2_ was absorbed by coal, van der Waals interaction accounted for about 36–48%, and electrostatic interaction accounted for about 52–64%, indicating that electrostatic interaction played a dominant role—that is, the higher the water content, the greater the proportion of electrostatic interaction. This interaction was different from the dry coal sample when CO_2_ was absorbed. When water-containing coal adsorbed CH_4_, van der Waals interaction energy accounted for about 99.5%, and electrostatic interaction accounted for about 0.5%. This result indicated that van der Waals interaction energy played a dominant role in adsorption, which was almost the same as that when dry coal samples adsorbed CH_4_.

## 5. Conclusions

By means of molecular simulation, we studied the pore structure characteristics of different gases and the influence rule and microscopic mechanism of different temperature, pressure, and water content on the adsorption performance of CO_2_ and CH_4_ gas by coal in the macromolecular structure model of noncohesive coal in the Chicheng Coal Mine. The main conclusions of this study are as follows:

(1) A three-dimensional macromolecular structure model of nonsticky coal in the Chicheng Coal Mine was constructed using molecular simulation software. Through geometric and dynamic optimization of the model, the final density of the model stabilized at 1.138 g/cm^3^, which was close to the actual coal density. The rationality of the constructed model was proved by comparing the adsorption results of CH_4_ between the model and the experiment. Based on this model, the accessible pore of CO_2_ and CH_4_ in dry coal samples was 2295.89 Å^3^ and 1550.92 Å^3^, respectively, by probe analysis.
(2) In the macromolecular structure model of dry coal, the higher the temperature was, the stronger the inhibition of gas adsorption capacity and interaction could be. The equivalent adsorption heat of CO_2_ decreased with the increase in temperature, the equivalent adsorption heat of CH_4_ changed little with the increase in temperature, the equivalent adsorption heat of CO_2_ decreased with the increase in pressure, and the equivalent adsorption heat of CH_4_ first increased and then decreased with the increase in pressure. Under the same conditions, the adsorption capacity, interaction energy, and adsorption heat of CO_2_ were all greater than that of CH_4_, and CO_2_ was more sensitive to temperature changes. The adsorption of the two gases in the coal molecular model was physical adsorption.
(3) The macromolecular structure model of water-bearing coal was established. In the macromolecular structure model of water-bearing coal, the higher the water content was, the smaller the adsorption capacity and interaction energy of the two gases were. The equivalent adsorption heat of CO_2_ and CH_4_ adsorbed in wet coal with different water content decreased with the increase in pressure and increased with the increase in water content.

## Figures and Tables

**Table 1:** Energy changes before and after model optimization.

State	Total Energy (kcal·mol^−1^)	Valence Electron Energy, *E*_V_				Nonhealthy Energy, *E*_N_	
		Bond (kcal·mol^−1^)	Angle (kcal·mol^−1^)	Torsion (kcal·mol^−1^)	Inversion (kcal·mol^−1^)	Van der Waals (kcal·mol^−1^)	Electrostatic (kcal·mol^−1^)
Initial state	236,115.853	1753.26	303.492	2625.519	23.275	230,432.156	−37.852
End state	2760.211	64.928	177.781	2567.972	21.346	111.852	−55.655

**Table 2:** Basic parameters of molecular structure.

Adsorption Mass Model	State	Total Energy (kcal·mol^−1^)	Valence Electron Energy (kcal·mol^−1^)	Nonhealthy Energy (kcal·mol^−1^)
CH_4_	Initial state	2.490521	2.178	0.000
	End state	0.223232	0.242	0.000
CO_2_	Initial state	216.391631	182.651	0.000
	End state	0.000023	0.000	0.000
H_2_O	Initial state	19.417523	19.043	0.000
	End state	0.000023	0.000	0.000

**Table 3:** Langmuir constant of adsorption of the two gases in the coal macromolecular model.

Type of Gas	Temperature (K)	*a*	*b*	Adj. R^2^
CH_4_	293.15	34.25	0.58	0.9917
	298.15	31.93	0.60	0.9911
	303.15	31.08	0.59	0.9953
	308.15	29.73	0.57	0.9917
	313.15	28.95	0.55	0.9926
CO_2_	293.15	44.54	0.55	0.9925
	298.15	41.95	0.87	0.9911
	303.15	40.64	0.81	0.9945
	308.15	38.09	0.85	0.9919
	313.15	36.09	0.79	0.9903

**Table 4:** Langmuir constant of adsorption of the two gases in coal macromolecular model.

Type of Gas	Content of Water (%)	*a*	*b*	Adj. R^2^
CO_2_	1	32.96	1.08	0.9905
	2	26.95	0.92	0.9890
	3	20.90	0.94	0.9851
	5	10.01	1.47	0.9721
CH_4_	1	24.13	0.77	0.9959
	2	19.04	0.89	0.9908
	3	15.62	0.88	0.9905
	5	8.60	0.95	0.9859

## Data Availability

The datasets used and/or analyzed during the current study are available from the corresponding author upon reasonable request.
